# Papillary renal cell carcinoma with metastatic laparoscopic port site and vaginal involvement: a case report

**DOI:** 10.1186/1752-1947-5-131

**Published:** 2011-04-01

**Authors:** Xue En Chuang, Hwai Liang Loh, Hong Gee Sim, Kah Leng Fong, Min-Han Tan

**Affiliations:** 1 Department of Medical Oncology, National Cancer Centre Singapore, Singapore; 2 Department of Pathology, Singapore General Hospital, Singapore; 3 Department of Urology, Singapore General Hospital, Singapore; 4 Department of Obstetrics and Gynaecology, Singapore General Hospital, Singapore

## Abstract

**Introduction:**

Laparoscopic port-site metastasis is a rare but well recognized outcome following surgery in urological cancers, with its etiology not clearly understood. Additionally, vaginal metastasis in clear cell renal cell carcinoma is rare, and has not been previously reported in the setting of papillary renal cell carcinoma.

**Case presentation:**

We present the case of a 71-year-old Chinese woman with metastatic type II papillary renal cell carcinoma with histologically verified vaginal involvement and a concurrent laparoscopic port-site metastasis. This was also associated with a unique constellation of widely disseminated metastatic sites, which include a local relapse, the peritoneum and the urethra.

**Conclusion:**

Laparoscopic port-site metastases are associated with the presence of advanced cancer with multiple sites of metastasis. We hypothesize from the findings of our report and background data that this phenomenon is more likely to be related to tumor factors rather than operative factors. We also present what is, to the best of our knowledge, the first reported case in the literature of vaginal and urethral metastasis and the second reported case of laparoscopic port-site recurrence.

## Background

Renal cell carcinoma is well known for its ability to metastasize widely to nearly every organ in the body. While vaginal metastases are very rare, with the mode of spread still currently obscure, it is critical to differentiate these metastases from primary vaginal carcinomas, which are rare and constitute approximately 2% of all malignant neoplasms of the female genital tract [1]. To date, all renal cell carcinoma (RCC) metastases to the vagina have been reported to be of the clear cell subtype. Additionally, up to September 2007, there were only 28 cases of port-site metastases involving urological malignancies reported. The etiology of port-site metastases has not been clearly established, though it appears to be multi-factorial [2].

## Case report

**Figure 1:**
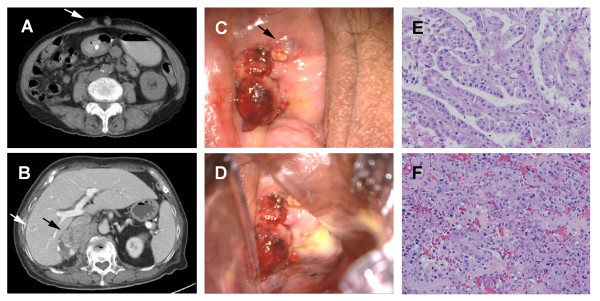
**Images of the metastatic papillary renal cell carcinoma (RCC)**. (A) Laparoscopic port-site metastasis (arrow); (B) local recurrence at the right renal bed (black arrow) and ring enhancing liver metastasis (white arrow); (C) a superficial mass representing tumor metastasis at the urethral orifice; (D) a similar tumor located at the right vaginal wall imaged on colposcopy, from which a biopsy was taken; (E) a hematoxylin and eosin stained histological section of the primary papillary RCC (20 × magnification); (F) a hematoxylin and eosin stained histological section of the metastatic vaginal lesion (20 × magnification).

A 71-year-old Chinese woman, with ischemic heart disease and a metallic stent and on prophylactic warfarin anticoagulation, presented to our institution with intermittent gross hematuria. A computed tomography (CT) scan showed an 8 cm mass in the upper pole of the right kidney, with no evidence of metastasis. Subsequently, a laparoscopic radical nephrectomy was performed, with the specimen bagged and removed through a lower abdominal incision. Histology results showed a type II papillary RCC, pT3A, nuclear grade 3, without sarcomatoid differentiation, with focal invasion of adjacent perirenal fat but with sparing of Gerota's fascia (Figure 1). Our patient relapsed six months after surgery, with local recurrence and multiple lesions in the lungs, liver, peritoneum, mesentery, iliac, and abdominal wall, as well as a laparoscopic port site metastasis (Figure 1). She was started on sunitinib 37.5 mg daily, and one week later, she presented with vaginal bleeding. Her international normalized ratio (INR) was 1.46. Colposcopy revealed a urethral mass as well as a hard nodular bleeding mass on the right vaginal wall (Figure 1). A vaginal biopsy yielded a papillary carcinoma, histologically consistent with the earlier diagnosis of RCC. She underwent palliative radiotherapy (30 to 36Gy in 10 to 12 fractions) and the bleeding was halted. She declined further systemic treatment, and died six months later, approximately one year after initial nephrectomy. Informed consent for this publication was obtained from her family.

## Discussion

Approximately 80 cases of vaginal metastasis from renal cell carcinoma have been reported to date, with indeterminate prognostic implications from conflicting case reports. After undergoing treatment, mostly in the form of nephrectomy and excision of the vagina lesion, some patients continue to live with no evidence of the disease, whereas others show rapid deterioration. Our report represents the first case of papillary RCC metastasizing to the vagina, with the second such report of a concurrent laparoscopic port-site metastasis. This case is highlighted because the patterns of metastasis for clear cell renal cell carcinoma and papillary renal cell carcinoma are recognized as being different [3]. Rare and unexpected sites of metastases in RCC are usually associated with the clear cell subtype.

Although immunohistochemical studies have suggested a common cellular origin for clear cell RCC and papillary RCC, there are distinct underlying genetic differences. Inactivation of the von Hippel-Lindau (*VHL*) gene occurs in patients with clear cell renal cell carcinoma in both the germline and somatic settings [4], whereas the underlying pathways that drive papillary RCC, particularly in the somatic setting, are less established. Reports indicating the identification of a familial cancer syndrome including type II papillary RCC from an underlying germline mutation in the fumarate hydratase (*FH*) gene and different activation patterns of cell cycle pathways between type I and type II papillary RCC have led to the role of metabolic signaling to be examined [5].

An anatomical explanation for vaginal metastasis has been advanced, supporting a predominant left-sided renal origin [6]. Consistent with the concept of retrograde venous spread as a mechanism of vaginal metastasis from renal cell carcinoma, retrograde flow of contrast medium from the left renal vein to the left ovarian vein, followed by filling of the ovarian and vaginal plexus has been demonstrated in patients with renal cell carcinoma [7]. Our case had a right-sided renal origin, but there was naturally widespread involvement of the systemic circulation including the lungs, which may account for this metastasis pattern.

Several hypotheses have been advanced to account for port-site metastasis, which is a recognized phenomenon [8-10], including contamination during laparoscopic surgery via surgical apparatus stained with exfoliated tumor cells, pneumoperitoneum or preferential growth of tumor cells at sites of high cellular proliferation during wound healing at the port site [11]. It is recognized that although port-site metastasis are rare, they normally occur in the presence of advanced disease [12]. Given that the single previous report of a port-site metastasis in type 2 papillary RCC had a similar profile of metastatic sites involving the peritoneum and liver in addition to the port site [13], our case report provides minor support for the hypothesis that port-site metastasis is related to tumor factors rather than operative factors.

## Conclusion

In summary, we report the first case of papillary renal cell carcinoma with metastasis to the vagina, with the second such report of a laparoscopic port-side metastasis. Our case report documenting a second port-site metastasis in a rare tumor provides support for the hypothesis that port-site metastases are related to tumor factors, and not operative factors.

## Consent

Written informed consent was obtained from the patient's next-of-kin for publication of this case report and any accompanying images. A copy of the written consent is available for review by the Editor-in-Chief of this journal.

## Competing interests

The authors declare that they have no competing interests.

## Authors' contributions

XEC and MHT wrote the report; HGS, KLF and MHT participated in the care of our patient; HLL provided an independent pathological review. All authors read and approved the final manuscript.

## References

[B1] Marchal F Leroux A Hoffstetter S Granger P Vaginal metastasis revealing colon adenocarcinoma Int J Colorect Dis 2006 21 861 862 10.1007/s00384-004-0734-x 15965696

[B2] Eng MK Katz MH Bernstein AJ Shikanov S Shalhav AL Zorn KC Laparoscopic port-site metastasis in urologic surgery J Endourol 2008 22 1581 1586 10.1089/end.2008.0329 18620507

[B3] Mai KT Landry DC Robertson SJ Commons AS Burns BF Thijssen A Collins J A comparative study of metastatic renal cell carcinoma with correlation to subtype and primary tumor Pathol Res Pract 2001 197 671 675 10.1078/0344-0338-00144 11700888

[B4] Maher ER Von Hippel-Lindau disease Curr Mol Med 2004 4 833 842 10.2174/1566524043359827 15579030

[B5] Yang XJ Tan MH Kim HL Ditlev JA Betten MW Png CE Kort EJ Futami K Furge KA Takahashi M Kanayama HO Tan PH Teh BS Luan C Wang K Pins M Tretiakova M Anema J Kahnoski R Nicol T Stadler W Vogelzang NG Amato R Seligson D Figlin R Belldegrun A Rogers CG Teh BT A molecular classification of papillary renal cell carcinoma Cancer Res 2005 65 5628 5637 10.1158/0008-5472.CAN-05-0533 15994935

[B6] Milathianakis CN Karamanolakis DK Massoud WA Roumier X Bogdanos IM Perrin P Vaginal metastases from renal cell carcinoma [in French] Prog Urol 2005 15 319 321 15999617

[B7] Mulcahy JJ Furlow WL Vaginal metastasis from renal cell carcinoma: radiographic evidence of possible route of spread J Urol 1970 104 50 52 5426709 10.1016/s0022-5347(17)61669-0

[B8] Dorrance HR Oien K O'Dwyer PJ Effects of laparoscopy on intraperitoneal tumor growth and distant metastases in an animal model Surgery 1999 126 35 40 10.1067/msy.1999.99056 10418590

[B9] Tan BJ Is carbon dioxide insufflation safe for laparoscopic surgery? A model to assess the effects of carbon dioxide on transitional-cell carcinoma growth, apoptosis, and necrosis J Endourol 2006 20 965 969 10.1089/end.2006.20.965 17144872

[B10] Neuhaus SJ Watson DI Ellis T Rofe AM Jamieson GG Influence of cytotoxic agents on intraperitoneal tumor implantation after laparoscopy Dis Colon Rectum 1999 42 10 15 10.1007/BF02235176 10211514

[B11] Patton MS Park KG Laparoscopic port site recurrence in the absence of intra-abdominal disease JR Coll Surg Edinb 2001 46 184 185 11478020

[B12] Zivanovic O Sonoda Y Diaz JP Levine DA Brown CL Chi DS Barakat RR Abu-Rustum NR The rate of port-site metastases after 2251 laparoscopic procedures in underlying malignant disease Gynecol Oncol 2008 111 431 437 10.1016/j.ygyno.2008.08.024 18929404

[B13] Masterson TA Russo P A case of port-site recurrence and locoregional metastasis after laparoscopic partial nephrectomy Nat Clin Pract Urol 2008 5 345 349 18490937 10.1038/ncpuro1127

